# FcεRI-HDAC3-MCP1 Signaling Axis Promotes Passive Anaphylaxis Mediated by Cellular Interactions

**DOI:** 10.3390/ijms20194964

**Published:** 2019-10-08

**Authors:** Misun Kim, Yoojung Kwon, Hyun Suk Jung, Youngmi Kim, Dooil Jeoung

**Affiliations:** 1 Department of Biochemistry, College of Natural Sciences, Kangwon National University, Chunchon 24341, Korea; misunjtl@naver.com (M.K.); kkwon89@kangwon.ac.kr (Y.K.); hsjung@kangwon.ac.kr (H.S.J.); 2 Institute of New Frontier Research, College of Medicine, Hallym University, Chunchon 24341, Korea; kym8389@hanmail.net

**Keywords:** Anaphylaxis, cellular interactions, FcεRI, HDAC3, MCP1, miRNAs

## Abstract

Anaphylaxis is an acute and life-threatening systemic reaction. Food, drug, aero-allergen and insect sting are known to induce anaphylaxis. Mast cells and basophils are known to mediate Immunoglobulin E (IgE)-dependent anaphylaxis, while macrophages, neutrophils and basophils mediate non IgE-dependent anaphylaxis. Histone deacetylases (HDACs) play various roles in biological processes by deacetylating histones and non-histones proteins. HDAC inhibitors can increase the acetylation of target proteins and affect various inflammatory diseases such as cancers and allergic diseases. HDAC3, a class I HDAC, is known to act as epigenetic and transcriptional regulators. It has been shown that HDAC3 can interact with the high-affinity Immunoglobulin E receptor (FcεRI), to mediate passive anaphylaxis and cellular interactions during passive anaphylaxis. Effects of HDAC3 on anaphylaxis, cellular interactions involving mast cells and macrophages during anaphylaxis, and any tumorigenic potential of cancer cells enhanced by mast cells will be discussed in this review. Roles of microRNAs that form negative feedback loops with hallmarks of anaphylaxis such as HDAC3 in anaphylaxis and cellular interactions will also be discussed. The roles of MCP1 regulated by HDAC3 in cellular interactions during anaphylaxis are discussed. Roles of exosomes in cellular interactions mediated by HDAC3 during anaphylaxis are also discussed. Thus, review might provide clues for development of drugs targeting passive anaphylaxis.

## 1. Introduction

Systemic anaphylaxis is an immediate acute reaction that is mediated by bioactive mediators, released mostly from mast cells [1,2]. These mediators can cause severe hypotension (low blood pressure), can decrease body temperature, and also increase β-hexosaminidase activity [3,4]. Mast cells and basophils are major cells that mediate IgE-dependent allergies and passive cutaneous anaphylaxis (PCA) [5,6]. Systemic anaphylaxis, PCA, and passive systemic anaphylaxis (PSA) involve the interaction between IgE and FcεRI. They also involve cellular interactions involving mast cells, basophils, macrophages and many other immune cells [7,8]. PSA can enhance the tumorigenic and metastatic potentials of cancer cells [4,7,9]. This implies that tumor microenvironments remodeled by allergic inflammation are characterized by cellular interactions between cancer cells and various immune cells.

Epigenetic modifications such as methylation and acetylation/deacetylation play critical roles in various allergic inflammatory diseases. The down regulation of DNA methyl transferase I (DNMT1) can induce PCA characterized by increased ear thickness and vascular leakage and enhanced angiogenic potential [10]. Tubastatin A, an inhibitor of histone deacetylase 6 (HDAC6), can attenuate airway inflammation by decreasing levels of IL-4, IL-5, and total inflammatory cell count [11]. Histone hyperacetylation caused by low expression level of HDACs in memory T cells contributes to eosinophilic airway inflammation [12]. T cell-specific deletion of HDAC1 can lead to airway inflammation accompanied by increased levels of Th2 cytokines [13]. Decreased expression of HDAC2 is associated with allergic airway inflammation [14] and PCA [15]. HDAC3 can interact with FcεRI and mediate allergic inflammations such as PCA and PSA [9,15]. Hyaluronic acid can attenuate allergic skin inflammation [16] and decrease the expression level of HDAC3 [17]. These reports suggest that HDACs play important roles in active and passive anaphylaxis. Since HDAC3 can bind to FcεRI and mediate passive anaphylaxis, mechanisms associated with HDAC3-mediated allergic inflammations merit further investigations.

In this review, we will focus upon the roles of HDAC3 in allergic inflammations such as PCA and PSA. Mechanisms of HDAC3-mediated PCA and PSA in association with the FcεRI signaling pathway and microRNA (miRNA) gene that forms a negative feedback loop with HDAC3 will be presented. The effect of monocyte chemoattractant protein 1 (MCP1), a chemokine regulated by HDAC3, on PCA and PSA, and cellular interactions during PCA and PSA, will be presented. Roles of exosomes in cellular interactions during passive anaphylaxis will also be presented.

## 2. Effector Cells, Effector Molecules, and Cellular Interactions in Anaphylaxis

**Figure 1:**
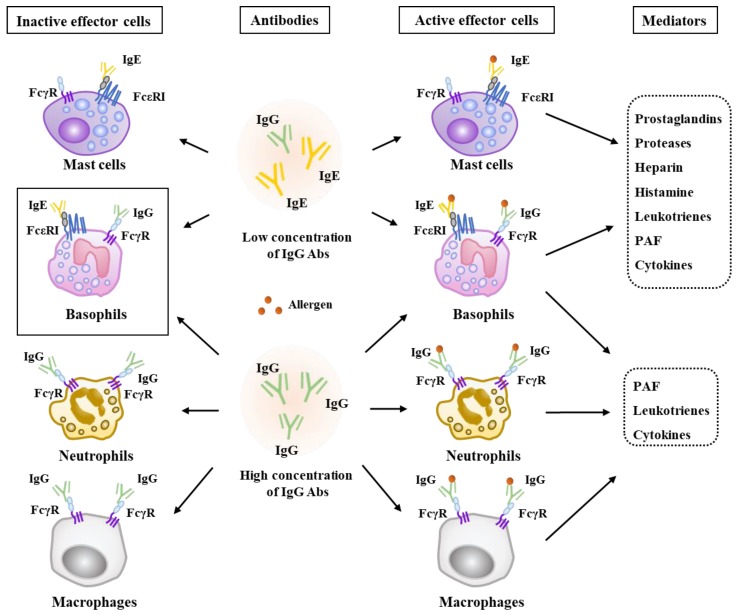
The mechanisms of antibody-mediated anaphylaxis. Allergen-specific Immunoglobulin E (IgE) antibodies and FcεRI-bearing effector cells play critical roles in anaphylaxis induced when concentrations of Immunoglobulin G (IgG) antibodies are low. Mast cells and basophils mediate IgE-mediated anaphylaxis. IgE-mediated anaphylaxis involves effector molecules including prostaglandins, histamine, leukotrienes, PAF, heparin, various cytokines and enzymes. The → arrows denote positive regulation.

Anaphylaxis is a serious allergic or hypersensitivity reaction that is rapid in onset. It can be life-threatening or fatal. IgE is the isotype found at the lowest concentration in circulation. However, IgE is found at a much higher level in patients with various allergic diseases. Allergen-specific IgE mediates active anaphylaxis and passive anaphylaxis, such as PCA and PSA [18,19,20,21]. Lactic acid (LA) abundant in inflamed tissues can suppress IgE-mediated hypothermia in mice undergoing PSA [22]. Omalizumab, an anti-IgE antibody, can prevent anaphylaxis in patients with mastocytosis [23,24] and exercise-induced anaphylaxis [25]. Mast cells express the high-affinity IgE receptor FcεRI and mediate IgE-dependent allergies and anaphylaxis, such as PCA and food-induced systemic anaphylaxis [2,6,26,27,28,29,30]. IgE can bind to FcεRI on the surfaces of blood basophils, tissue-resident mast cells and other cell types, including neutrophils, eosinophils, both monocytes and dendritic cells and platelets (Figure 1). Transfer of antigen-specific IgE into naive mice can sensitize animals to undergo anaphylaxis on subsequent exposure to that allergen. Such IgE-mediated cutaneous and systemic anaphylaxis is abrogated in mice that are lacking the high-affinity IgE receptor FcεRI [31], as well as in mast cell–deficient mice [18,32,33]. These reports indicate that both IgE and FcεRI play important roles in anaphylaxis.

Bivalent or multivalent allergens can induce cross-linking of FcεRI-bound IgE, leading to the activation of mast cells and basophils with immediate release of preformed mediator, such as histamine and various proteases. It also leads to de novo syntheses of many inflammatory mediator such as certain leukotrienes (LTs), prostaglandins and cytokines [19,27,28,34] (Figure 1). Mast cells and basophils can produce histamine during anaphylaxis (Figure 1). IgE binds to allergens and then sensitizes mast cells through the Fc receptor, resulting in the secretion of various pro-inflammatory mediators. Histamine, a well-known mediator of anaphylaxis, can induce the vascular permeability accompanied by PCA [35]. Mast cells can mediate PCA via heparin formation [36,37] (Figure 1). CysLTs (LTB4, LTC4 and LTD4) synthesized from arachidonic acid by mast cells and basophils can cause bronchoconstriction in PCA and asthma. Adenine inhibits IgE-dependent PCA and blocks the production of LTB4 by mast cells [38]. The PAF (platelet activating factor), a phospholipid-derived mediator, is released by neutrophils during Immunoglobulin G (IgG)-mediated drug-induced anaphylaxis. It mediates drug-induced anaphylaxis [39,40] (Figure 1). PAF induces degranulation in lung mast cells and peripheral blood (PB)-derived mast cells [41].

Anaphylatoxins such as C3a, C4a and C5a can also mediate anaphylaxis. Intradermal injection of C3a or C5a induces IgE-dependent mast cell degranulation and allergic skin inflammation [42]. C5aR1 mediates IgE-mediated food allergy through its regulation of allergen-specific IgE production and FcεR1-mediated mast cells degranulation [43]. However mechanisms of systemic and passive anaphylaxis mediated by these effector molecules remain largely unknown.

Some patients with anaphylaxis display low levels of IgE. This implies the presence IgE-independent anaphylaxis. IgE and IgG1 signaling are both necessary for anaphylactic responses to peanut [44,45]. IgG subclass determines pathways of PSA [46]. Allergen-specific IgG antibodies contribute to anaphylaxis when the allergen is an abundant circulating large molecule such as a therapeutic antibody (Figure 1). IgG-mediated anaphylaxis typically requires a much larger dose of allergen than IgE-mediated anaphylaxis. The FcγRIII in macrophages can mediate allergen-induced anaphylaxis [47]. The anti-IgG antibody activates neutrophil and contributes to drug-induced anaphylaxis. Monocytes and macrophages express high levels of activating FcγRs [45,48] and respond to anaphylatoxins [49]. Studies in mice have shown that the depletion of monocytes and macrophages by clodronate liposomes can reduce IgG-mediated active systemic anaphylaxis [50]. Macrophage are necessary for IgG-mediated passive systemic anaphylaxis [51] (Figure 1). These reports suggest that neutrophils, monocytes and macrophages also play important roles in IgG-mediated anaphylaxis.

Basophils, neutrophils, and monocytes/macrophages play critical roles in anaphylaxis induced by allergens that form immune complexes in the presence of high concentrations of IgG antibodies. Histamine strongly mediates anaphylaxis in human subjects. Leukotrienes, the platelet activating factor (PAF) and heparin mediate anaphylaxis in the mouse, but their roles in human subjects are not clear. Basophils may mediate both IgE and IgG-dependent anaphylaxis.

Morin, a natural flavonoid, can inhibit IgE-mediated allergic response by decreasing levels of Th2-cytokines, NF-κB, and IgE [52]. Curcumin can suppresses intestinal anaphylaxis by inhibiting NF-κB in bone marrow-derived mast cells [53]. Anaphylaxis involves impairing tolerance induced by TGFβ1 and regulatory T cells. TGFβ1 and FOXP3 can induce oral tolerance to food allergens [54,55]. TGFβ1 inhibits ovalbumin-promoted anaphylaxis by suppressing IL-4 production and GATA-3 expression [56]. Food allergen-induced anaphylaxis is mediated by Th2 cytokines such as IL-4, IL-5 and IL-13 [57,58]. Food allergen-induced anaphylaxis involves TGFβ-dependent Treg-cell suppression by mast cell activation [59]. Glycomacropeptide (GMP), a novel inhibitor of anaphylaxis, can increase the production of TGFβ1, attenuate mast cell activation by allergen, and suppress the secretion of histamine in the rat model of anaphylaxis [59]. These reports suggest that PSA mediated by IgE is closely associated with suppression of functions of FOXP3^+^ Tregs. In atopic dermatitis, the expression of FoxP3, a marker of Treg cells, is decreased [60]. This suggests that anaphylaxis and atopic dermatitis might share molecular features.

Mast cells are major residents of solid tumors. They can interact with endothelial cells to contribute to angiogenesis in multiple myelomas [61]. Mast cells activated by IL-33 produced by tumor epithelium can promote the growth of gastric cancers. Inactivation of mast cells can suppress the proliferation of tumor-associated macrophages and reduce cancer cell proliferation [62]. These reports imply that anaphylaxis is mediated by cellular interactions involving mast cells. PCA and PSA both involve interactions among mast cells and macrophages [9]. Extracellular vesicles (exosomes) are membranous vesicles of 30–100 nm diameter secreted by cells into the extracellular environment. Exosomes are formed by the fusion of multivesicular bodies with the cell membrane. The exosome can mediate cellular interactions among mast cells, macrophages and cancer cells [4,61,62]. Mechanisms of cellular interactions mediated by these exosomes during anaphylaxis remain largely unknown.

## 3. HDACs (Histone Deacetylases)

Acetylation and deacetylation on lysines of histones and non-histone proteins regulate gene expression by modifying chromatin structures. Histone deacetylases (HDACs) can remove acetyl groups on the N-terminal lysines of histones, leading to transcriptional repression [63]. Eighteen HDAC enzymes have been identified in mammalian cells. Class I HDACs (HDACs 1, 2, 3 and 8), class II HDACs (HDACs 4, 5, 6, 7, 9 and 10), and class IV HDAC (HDAC 11) require Zn2^+^ as a cofactor in their active sites. Class III HDACs consist of sirtuins, which use NAD^+^ as a cofactor for their enzyme activities. Sirtuins (SIRT 1–7) are widely expressed in human tissue. They shuttle between the nucleus and the cytoplasm (SIRT-1 and SIRT-2). SIRT-3, SIRT-4 and SIRT-5 are present in the mitochondria. SIRT-6 and SIRT-7 are present in the nucleoli [64]. HDAC1 and HDAC2 are present in the nucleus while HDAC3 and HDAC8 shuttle between the nucleus and cytoplasm [65]. Unlike many other HDACs, HDAC3 shows ubiquitous expression [66]. HDACs also deacetylate non-histone proteins [67,68,69,70]. Transcription factors, signal transduction molecules and chaperone proteins can serve as substrates of HDACs [71,72,73]. Class I HDACs play important roles in various biological events through their non-histone substrates. For example, HDAC3 deacetylate myocyte enhancer factor 2 (MEF2) which plays a critical role in muscle development [74]. The nuclear receptor corepressor SMRT can enhance the deacetylase activity of HDAC3 toward MEF2 [74].

## 4. HDACs Inhibitors and Allergic Inflammatory Diseases

Histone acetylation and deacetylation play an important role in the regulation of inflammatory genes associated with allergic inflammations such as allergic rhinitis and asthma [75,76]. TSA, a non-selective inhibitor of class I and class II, decreases the expression of Th2 cytokines (IL-4, IL-15, and IL-13) and the IgE level in the mouse model of asthma [77]. The airway epithelium of asthmatic subjects shows increased levels of histone H3K acetylation [78]. HDAC inhibitors can suppress inflammatory diseases such as acute-on-chronic liver failure by regulating acetylation status of NF-κB [79]. HDAC inhibitors can suppress TNFα-induced NF-κB activation [80]. NF-κB activation is seen in an experimental model of asthma [81]. TLR4/MyD88/ROS/NF-κB signaling mediates mast cell-dependent allergic asthma [82]. Both PCA and PSA require activation of NF-κB [83]. Inhibitors of HDACI/II can decrease levels of IL-6 and TNF-α in macrophages from patients with rheumatoid arthritis [84]. The expression and activity of enzymes that regulate acetylation and deacetylation are linked to airway diseases [85]. Sodium butyrate, an inhibitor of HDACs, can decrease levels of ovalbumin-specific IgE and improve clinical symptoms and nasal mucosa epithelial morphology in a mouse model of allergic rhinitis [86]. PCA reactions can increase expression levels of inflammatory cytokines via the activation of PI3K/AKT, MAPK and NF-κB [87]. HDAC inhibitors such as TSA can inhibit PCA and TpCR [15]. Sirt1, a class III HDAC, can suppress FcεRI-stimulated mast cell activation and passive anaphylaxis. The mast cell-specific knockout of Sirt1 shows increased mast cell activation both in vitro and in vivo [88,89]. These reports suggest that HDACs play important roles in allergies and anaphylaxis.

## 5. A Negative Regulatory Role of HDAC2 in Allergic Inflammation

Histone deacetylase 2 (HDAC2) mRNA is decreased in bronchoalveolar lavage cells from steroid-resistant patients with asthma. Glucocorticoid receptor β can decrease the expression of HDAC2 by inhibiting glucocorticoid response elements in promoter sequences of HDAC2 [90]. In the BALB/C mouse model of ovalbumin-induced steroid-insensitive allergic airway disease, viral infection can increase the expression level of miRNA-21 (miR-21), which decreases the expression level of HDAC2 by activating PI3 kinase [91]. Patients with asthma show reduced expression of HDAC2 compared to healthy controls [92]. Histone acetyl transferase (HAT) activity is increased in patients with asthma treated with inhaled steroids [92]. HDAC2 can suppress house dust mite (HDM)-induced allergic inflammation by decreasing the expression of IL-17A [93]. Inhibition and silencing of HDAC2 can block TGFβ1 signaling in the airway epithelium [94]. Given the fact that TGFβ1 confers oral tolerance to food allergens [54,55], HDAC2 might act as a negative regulator of allergies and anaphylaxis. Taken together, these reports implicate the roles of HDACs including HDAC2 in allergies and anaphylaxis.

## 6. HDAC3 Mediates Anaphylaxis

HDAC3 binds to corepressor proteins that do not form complexes with other class I HDACs. The HDAC3 complex contains a nuclear receptor corepressor such as N-CoR. Deletion analysis of N-CoR has revealed that HDAC3 can bind to multiple N-CoR regions [95]. HDAC3 acts as a repressor of the PD-L1 and HDAC3 inhibitor, resulting in lymphoma regression [96]. HDAC3 can regulate inflammatory gene expression in rheumatoid arthritis fibroblast-like synoviocytes [97]. Inhibition of HDAC3 can decrease LPS-induced expression of inflammatory cytokine genes such as TNF-α, IL-1β, and iNOS [98]. Loss of HDAC3 inhibits the activation of inflammatory genes in macrophages in response to LPS [99]. RGFP966, a selective inhibitor of HDAC3, displays anti-inflammatory effects in response to LPS/IFNγ in RAW 264.7 macrophages by increasing the expression of anti-inflammatory gene IL-10 via NF-κB [100]. These reports imply a role of HDAC3 in allergies and anaphylaxis.

Src can bind to HDAC3 at the plasma membrane. HDAC3 is a substrate of Src [67]. FcεRI signaling involves the activation of Src family kinases such as Lyn [87] and Syk [101]. This implies that HDAC3 might mediate FcεRI signaling in allergic inflammations.

DNA methyltransferase I (DNMT1) is a negative regulator of triphasic cutaneous reaction (TpCR) and PCA by increasing the expression of HDAC3 [10]. TGase II is increased in the BALB/C mouse model of PCA. It mediates PCA and atopic dermatitis [102]. TGase II binds to NF-κB and directly regulates the expression of HDAC3 by binding to promoter sequences of HDAC3 [102]. These reports imply that HDAC3 can regulate allergic inflammation such as passive anaphylaxis by mediating FcεRI signaling.

**Figure 2:**
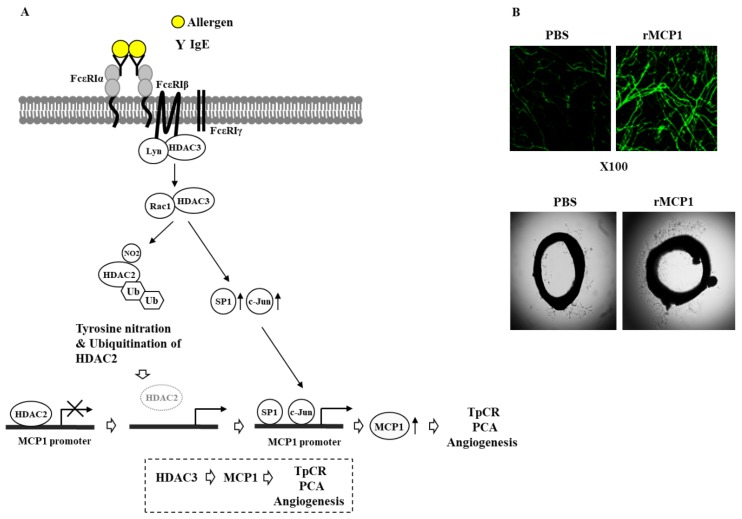
Histone deacetylase 3 (HDAC3) binds to FcεRI and mediates a triphasic cutaneous reaction (TpCR) and passive cutaneous anaphylaxis (PCA). (**A**) In the BALB/C mouse model of TpCR and PCA, HDAC3 binds to FcεRI. HDAC3, through the interaction with rac1, leads to the tyrosine nitration of HDAC2. HDAC3 also induces ubiquitination of HDAC2 to decrease the expression of HDAC2. HDAC3 increases the expression of SP1 and C-Jun, which bind to the promoter sequences of monocyte chemoattractant protein 1 (MCP1) to increase the expression of MCP1. In the absence of allergen stimulation, HDAC2 binds to the promoter sequences of MCP1 to suppress the expression of MCP1. (**B**) Mouse recombinant MCP1 protein displays angiogenic potential based on whole mount staining employing anti-PECAM antibody (upper panel) and aortic ring formation assays (lower panel). In conclusion, FcεRI-HDAC3-MCP1 signaling mediates TpCR, PCA and angiogenesis. The ↑ arrows denotes increased expression level and → arrows denote activation of transcription and positive regulation. Hollow arrows denote positive regulation.

Both TpCR and PCA can increase the expression of HDAC3 (Figure 2A). Promoter methylation may affect expression levels of HDAC3. It would also be necessary to identify transcription factors that can regulate the expression of HDAC3. HDAC3 can bind to FcεRI and mediate anaphylaxis (Figure 2A). Trichostatin A (TSA), an inhibitor of HDAC(s), can disrupt the interaction between FcεRI and HDAC3 [15]. HDAC3 can decrease the expression of HDAC2 in the presence of allergen stimulation through its interaction with rac1 (Figure 2A). Rac1 promotes proteasomal degradation of p21 [103] and SWI/SNF protein BAF60b [104]. Down-regulation of HDAC2 can be due to posttranslational modification such as ubiquitination [105,106]. The down-regulation of HDAC2 by allergen stimulation may result from tyrosine nitration and ubiquitination [15] (Figure 2A). In mouse models of systemic anaphylaxis and atopic dermatitis, an increased level of MCP1 is accompanied by mast cell degranulation and elevated levels of Th2/Th17/Th1 cytokines [107]. HDAC2 can bind to the promoter sequences of MCP1 to decrease the expression of MCP1 in the absence of allergen stimulation [15] (Figure 2A). Since HDAC3 decreases the expression level of HDAC2, HDAC3 might regulate the expression level of MCP1.

HDAC3 can directly regulate the expression of MCP1 based on ChIP assays [15] (Figure 2A). HDAC3 and MCP1 are necessary for PCA accompanied by ear swelling, enhanced vascular permeability and angiogenesis [15] (Figure 2A). Recombinant MCP1 can enhance β-hexosaminidase activity and increase the amount of histamine released. It also shows angiogenic potential [15] (Figure 2B). We have shown that atopic dermatitis, PCA and PSA display common molecular features such as activation of FcεRI signaling, increased secretion of Th2 cytokines and increased amount of histamine released [60]. Thus, MCP1 might mediate atopic dermatitis and passive anaphylaxis. Our results show that HDAC3 can mediate PCA and PSA through its effects on FcεRI signaling and MCP1 expression.

## 7. HDAC3, miRNAs, MCP1, and Cellular Interactions in Anaphylaxis

**Figure 3:**
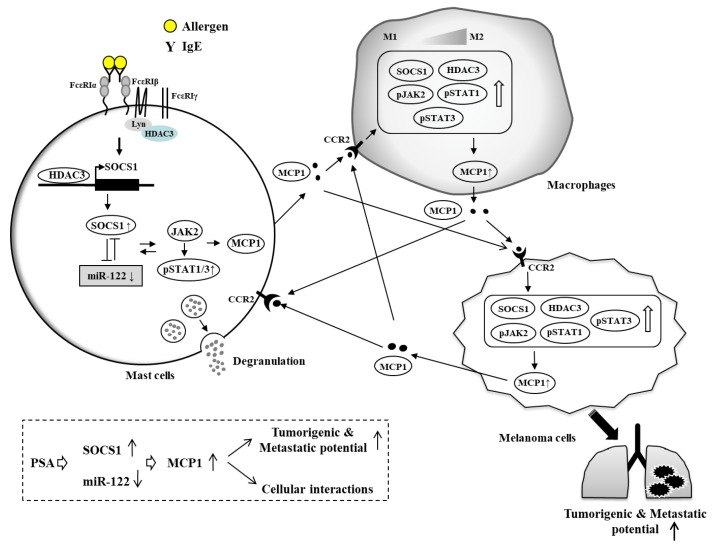
MCP1 mediates cellular interactions in passive cutaneous anaphylaxis (PCA) and passive systemic anaphylaxis (PSA) regulated by the miR-122-SOCS1 negative feedback loop. Allergen stimulation activates FcεRI signaling and increases the expression of SOCS1. HDAC3 binds to the promoter sequences of SOCS1 to increase the expression of SOCS1. Allergen stimulation decreases the expression of TGFβ1 and miR-122a-5p. In this study, TGFβ1 exerts a negative effect on allergic inflammation [7]. MiR-122a-5p and SOCS1 form a negative feedback loop. JAK2/STA3 signaling, activated by SOCS1, is responsible for the increased production and secretion of MCP1. MCP1, through CCR2, mediates cellular interactions during anaphylaxis. MCP1 also enhances the tumorigenic and metastatic potential of melanoma cells. The → arrows denote activation of transcription and ↑ arrows denotes increased expression level/ increased characteristics. Hollow arrows denote positive regulation. Both side T-bar arrows denote cross inhibition. SOCS1, Suppressor of cytokine signaling1; JAK2, Janus kinase2; MCP1, Monocyte chemoattractant protein-1; STAT, Signal transducer and activator of transcription; HDAC3, Histone deacetylase 3; CCR2, c-c chemokine receptor type 2; PSA, Passive systemic anaphylaxis.

MicroRNAs (miRNAs) target multiple genes, thereby playing various roles in biological processes. *MiR-20a* can inhibit the expression of pro-inflammatory cytokines that mediate allergic inflammation [108]. Increased expression of *miR-142-3p* is correlated with aberrant wnt signaling in human and murine asthma [109]. *MiR-26a* and *miR-26b* target COX-2 and regulate PCA and PSA and tumorigenic potential of melanoma cells enhanced by PSA [110]. *MiR-155* can mediate IL-10-promoted passive systemic anaphylaxis by targeting SOCS [111]. Anaphylactic shock can increase the expression of SOCS1 known to regulate anaphylactic shock viscera injury processes [112]. SOCS1 can bind to FcεRI and is necessary for tumorigenic and metastatic potential of cancer cells enhanced by PSA (Figure 3). This indicates role of SOCS1 in passive anaphylaxis. SOCS1 forms a negative feedback loop with *miR-122a-5p* and regulates cellular interactions involving cancer cells, mast cells and macrophages during PSA [7] (Figure 3). The *MiR-122a-5p*-SOCS1 axis regulates JAK/STAT signaling and the expression of MCP1 [7]. SOCS1 regulates the expression of HDAC3. It is necessary for the decreased production of TGFβ1 in PSA [7]. TGFβ1 exerts a negative effect on allergic inflammation in vitro [7]. The *MiR122a-5p*-SOCS1 axis also regulates the clinical symptoms and molecular features of atopic dermatitis [60]. These reports suggest roles of miRNAs in allergies and allergic anaphylaxis.

**Figure 4:**
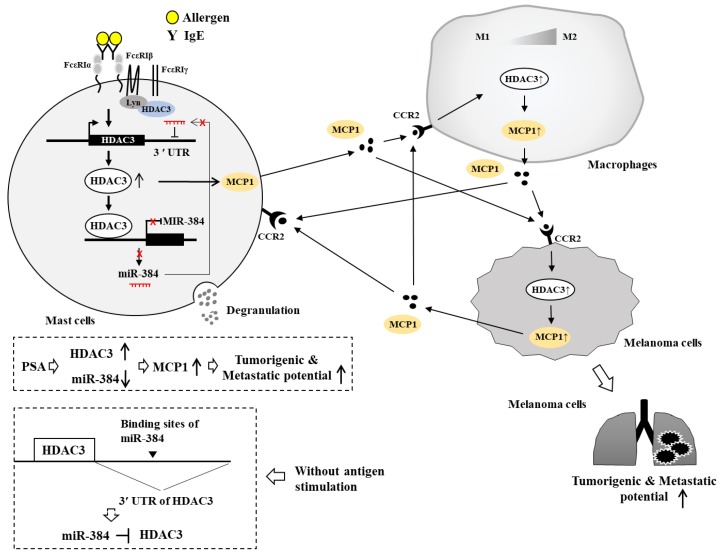
HDAC3-miR-384 negative feedback loop regulates PCA and PSA and cellular interactions during PCA and PSA. PSA increases the expression of HDAC3 and induces the activation of FcεRI signaling. HDAC3 binds to the promoter sequences of miR-384 to decrease the expression of miR-384. TargetScan predicts miR-384 as a negative regulator of HDAC3. In the absence of allergen stimulation, miR-384 binds to the 3′UTR (untranslated region) of HDAC3 to decrease the expression of HDAC3. Thus, HDAC3 and miR-384 form a negative feedback loop. HDAC3 increases the expression of MCP1, which mediates cellular interactions and enhances the tumorigenic and metastatic potential of melanoma cells. MiR-384 negatively regulates PCA and PSA and the tumorigenic and metastatic potential of melanoma cells enhanced by PSA. Allergen-stimulated mast cells and melanoma cells promote a differentiation of M2 macrophages (TAM). M2 macrophages display a higher expression of CD163, but lower expressions of inducible nitric oxide synthase (iNOS) than M1 macrophages. Allergen-activated macrophages (M2) also activate mast cells and melanoma cells. Cellular interactions in this study were investigated by co-culture experiments. The ↑ arrows denote increased expression level/ increased characteristics and ↓ arrows denote decreased expression level. Hollow arrows denote positive regulation and T-bar arrows denote negative regulation. MCP1, Monocyte chemoattractant protein-1; HDAC3, Histone deacetylase 3; CCR2, c-c chemokine receptor type 2; PSA, Passive systemic anaphylaxis.

We investigated the role of HDAC3 in anaphylaxis in association with miRNAs. TargetScan analysis predicted that HDAC3 was a target of *miR-384*. HDAC3 that is increased by allergic inflammation could bind to promoter sequences of *miR-384* (Figure 4). Luciferase activity assays showed direct regulation of HDAC3 by *miR-384* in the absence of allergen stimulation (Figure 4). HDAC3 might also affect the expression of transcription factors known to regulate expression of *miR-384*. *MiR-384* can bind to the 3′ UTR of HDAC3 to decrease the expression of HDAC3 (Figure 4). Thus, HDAC3 and *miR-384* can form a negative feedback loop to regulate allergic inflammations such as PCA and PSA.

Cytokine array analysis has revealed that MCP1, among various cytokines and chemokines, is significantly decreased by the down-regulation of HDAC3 [9]. HDAC3 and MCP1 are necessary for the tumorigenic and metastatic potential of melanoma cells enhanced by PSA (Figure 4). Mast cells activated during PCA promote angiogenesis via FcεRI-EGFR cross talk [113]. IL-33 produced by mast cells can mediate PCA [114]. It is necessary for IgE-mediated food-induced anaphylaxis [115]. Mast cells can enhance angiogenesis via MCP1 [116,117]. Inflammatory mast cells can promote angiogenesis during squamous epithelial carcinogenesis via mast cell-specific proteases MCP-4 and MCP-6 [118]. Mast cells enhance the tumorigenic potentials of cancers [7,119]. Mast cells activated by tumor-derived IL-33 can promote gastric cancer growth by mobilizing macrophage [62]. Mast cell-derived hypoxia-inducible factor-1 is necessary for promoting melanoma growth [120]. Mast cell-derived angiopoietin-1 plays a critical role in the growth of plasma cell tumors [121]. Thus, this tumorigenic potential of cancer cells enhanced by passive anaphylaxis may result from interactions among cancer cells, mast cells and other various immune cells. Soluble mediators may mediate these cellular interactions to regulate tumorigenic potential enhanced by passive anaphylaxis.

We hypothesize that MCP1 might mediate cellular interactions during PCA and PSA. MCP1 can bind to CCR2 and mediate cellular interactions among mast cells, macrophages, and melanoma cells during allergic inflammation [9] (Figure 4).

Based on the fact that HDAC3 plays a critical role in the activation of mast cells, HDAC3 may mediate cellular interactions among mast cells, endothelial cells, monocytes and macrophages during allergic inflammations such as PCA and PSA. HDAC inhibitors can suppress interactions between monocytes and endothelial cells [122]. Taken together, these reports suggest roles of HDAC3, MCP1 and miRNAs in anaphylaxis mediated by cellular interactions. Further studies are needed to identify molecules regulated by HDAC3 for a better understanding of cellular interactions mediated by HDAC3 and MCP1 during anaphylaxis.

## 8. Future Directions

**Figure 5:**
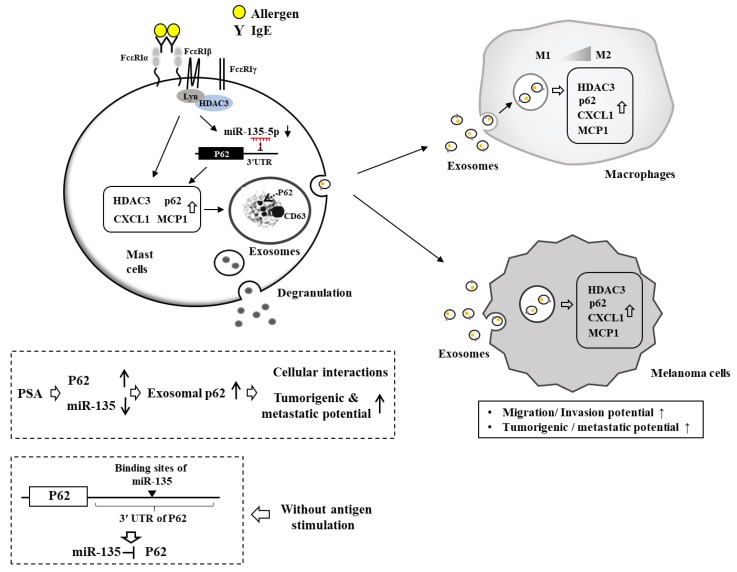
Exosomes mediate cellular interactions during PCA and PSA. Allergen stimulation induces the interaction between FcεRI and HDAC3. The expression of p62, a selective receptor of autophagy, is increased by allergen stimulation. The expression levels of HDAC3 and MCP1 are also increased. P62 binds to the promoter sequences of miR-135-5p to decrease the expression of miR-135-5p. In the absence of allergen stimulation, miR-135-5p binds to the 3′ UTR of P62 to decrease the expression of p62. P62 is present in the exosomes of allergen-stimulated mast cells and mediates cellular interactions during PCA and PSA. P62-containig exosomes promote differentiation of M2 macrophages. M2 macrophages, activated by exosomes, display higher expressions of CD163, but a lower expression of iNOS than the M1 macrophages. P62 increases expression levels of HDAC3 and MCP1 in macrophages. P62-containing exosomes also enhance tumorigenic and metastatic potential of melanoma cells. CD63 is a surface marker of exosomes. The presence of p62 within the exosomes was confirmed by immuno-electron microscopy. Arrow indicates p62 in the exosomes. The T-bar arrows denote negative regulation and hollow arrows denote positive regulation. The ↑ arrows denote increased expression level/ increased characteristics and ↓ arrows decreased expression level.

Exosomes of allergen-stimulated mast cells can activate macrophages and cancer cells (Figure 5). These exosomes may mediate interactions among cancer cells and various immune cells within the tumor microenvironment. It will be necessary to examine the expression profiles of cytokines and miRNAs in the exosomes of mast cells before and after allergen stimulation. These cytokines and miRNAs can mediate cellular interactions during PCA and PSA.

It is assumed that miRNAs can be developed as drugs targeting passive anaphylaxis. Exosomal miRNAs regulated by HDAC3 can also be developed as drugs targeting passive anaphylaxis. It will also be interesting to examine the presence of HDAC3 and/or MCP1 in exosomes of the serum of BALB/C mouse under PCA and PSA.

Allergic airway inflammation is accompanied by mTOR-mediated autophagy [123]. ATG7, a marker of autophagy, is necessary for degranulation in mast cells [124]. B cell autophagy can aggravate experimental asthma [125]. PCA reactions are accompanied by an enhanced autophagic flux which is necessary for the degranulation in mast cells [126]. Autophagy inhibitor chloroquine can attenuate clinical symptoms associated with allergic asthma [127]. P62, a selective receptor of autophagy, is present in exosomes of allergen-stimulated mast cells. It can mediate cellular interactions during PCA and PSA (Figure 5). P62 can increase expression levels of HDAC3 and MCP1 in the presence of allergen stimulation (Figure 5). Invariant natural killer T (iNKT) cell development requires autophagy which is dependent on HDAC3 [128]. Based on the fact that HDAC3 regulates PCA and PSA, HDAC3 may affect cellular interactions during PCA and PSA through its effect on autophagic flux. Thus, further studies are needed to examine the effect of HDAC3 on autophagic flux during anaphylaxis.

HDAC3 interacts with FcεRI and mediates PCA and PSA (Figure 2). Identification of the domain of HDAC3 necessary for its interaction with FcεRI may provide clues to the development of therapeutics targeting PCA and PSA. Peptides corresponding to the domain of HDAC3 necessary for an interaction of HDAC3 with FcεRI can be developed as drugs targeting allergies and anaphylaxis. Small molecules that fit with the structure of the HDAC3 protein could be developed as drugs targeting PCA and PSA, and food-induced systemic anaphylaxis.

Mechanisms associated with the role of HDAC3 in PCA and PSA remain largely unknown. Although we have identified *miR-384* as a negative regulator of HDAC3, further identification of miRNAs that form negative feedback loops with HDAC3 will be necessary for the better understanding of HDAC3-mediated anaphylaxis. TargetScan analysis will give information on miRNAs that can regulate expression of HDAC3. These miRNAs may regulate PCA and PSA. They can be employed as drugs for allergies and passive anaphylaxis such as PCA and PSA. Non-selective HDAC inhibitors have been developed for the treatment of allergic diseases. However, most of these inhibitors have limited clinical values. HDAC3-specific inhibitors would be useful for the treatment of allergies, PCA, PSA and food-induced systemic anaphylaxis.
